# Pregnancy audit in the PATH (Psychosis Prevention, Assessment and Treatment in Hertfordshire) early intervention in psychosis service

**DOI:** 10.1192/bjo.2021.144

**Published:** 2021-06-18

**Authors:** Jessica Kershaw, Zohra Taousi, Hildah Jiah, Giovanni Borghini, Debbi Knight, Keith Brown

**Affiliations:** Hertfordshire Partnership University NHS Foundation Trust

## Abstract

**Aims:**

To identify whether staff from the PATH Early Intervention in Psychosis (EIP) Service routinely ask female service users of child bearing age about their plans for pregnancy, whether risks of medication in pregnancy are routinely discussed and whether contraception is routinely discussed.

**Method:**

In May 2019, a report was run capturing all female PATH service users of child bearing age (16-50 years) who were on the pathway at this time. This totalled 177 service users, all of whom were included in the sample. We used the search terms “pregnant”, “pregnancy”, “having children”, “contraception”, “conceive”, “baby”, “conception”, “miscarriage”, “abortion”, “IVF”, “still born” to interrogate the patient records . Auditors searched case notes, clinic letters, recent physical health assessment and recent wellbeing plan for evidence as to whether staff had asked about pregnancy plans, contraception, offered a referral to the Community Perinatal Team, and discussed risks about medication in pregnancy.

**Result:**

Of the 177 service users, 34 were asked whether they had plans for pregnancy (19%). Of the 177 service users, 28 were given advice regarding contraception (16%). Of the 34 service users who were asked about pregnancy plans, 27 did have plans for pregnancy. Of these 27 service users, 15 were offered a referral to the Community Perinatal Team (56%). Of the 27 service users who did have plans for pregnancy, 12 received advice and or information about risks of antipsychotic medication in pregnancy (44%).

**Conclusion:**

It is clear that PATH staff are not routinely having discussions with female service users of child bearing age about their plans for pregnancy or contraception; this audit has identified that this occurs in less than 20% of cases. Of service users that did have plans for pregnancy, only 56% were offered a referral to the Community Perinatal Team; we should strive for this to be 100% so service users can access specialist support and advice. Work is underway to include information on pregnancy in the PATH service information leaflet to ensure women referred to PATH expect staff to ask them about their plans for pregnancy and contraception. Questions about pregnancy planning and contraception are to be embedded in the Trust's Physical Health Assessment care document to act as a prompt for staff. Finally, the topics of pregnancy and contraception in women with psychosis have been incorporated into the PATH physical health training programme which will be delivered with support from the Community Perinatal Team.

